# Dual-Positive MPO- and PR3-ANCA-Associated Vasculitis Following SARS-CoV-2 mRNA Booster Vaccination: A Case Report and Systematic Review

**DOI:** 10.3390/vaccines10050653

**Published:** 2022-04-21

**Authors:** Eva Baier, Ulrike Olgemöller, Lorenz Biggemann, Cordula Buck, Björn Tampe

**Affiliations:** 1 Department of Nephrology and Rheumatology, University Medical Center Göttingen, 37085 Göttingen, Germany; eva.baier@med.uni-goettingen.de; 2 Department of Cardiology and Pneumology, University Medical Center Göttingen, 37085 Göttingen, Germany; ulrike.olgemoeller@med.uni-goettingen.de (U.O.); cbuck@med.uni-goettingen.de (C.B.); 3 German Center for Cardiovascular Research (DZHK), Partner Site Göttingen, 37099 Göttingen, Germany; 4 Institute of Diagnostic and Interventional Radiology, University Medical Center Göttingen, 37085 Göttingen, Germany; lorenz.biggemann@med.uni-goettingen.de

**Keywords:** booster vaccination, SARS-CoV-2, systemic vasculitis, ANCA-associated vasculitis, pulmonary hemorrhage

## Abstract

As the coronavirus disease 2019 (COVID-19) pandemic is ongoing, and new variants of severe acute respiratory syndrome coronavirus type 2 (SARS-CoV-2) are emerging, vaccines are needed to protect individuals at high risk of complications and to potentially control disease outbreaks by herd immunity. After SARS-CoV-2 vaccination, antineutrophil cytoplasmic antibody (ANCA)-associated vasculitis (AAV) presenting with a pulmonary hemorrhage has been described. Previous studies suggested that monocytes upregulate major histocompatibility complex (MHC) II cell surface receptor human leukocyte antigen receptor (HLA-DR) molecules in granulomatosis with polyangiitis (GPA) patients with proteinase 3 (PR3)- and myeloperoxidase (MPO)-ANCA seropositivity. Here, we present a case of new-onset AAV after booster vaccination with the Pfizer-BioNTech SARS-CoV-2 mRNA vaccine. Moreover, we provide evidence that the majority of monocytes express HLA-DR in AAV after SARS-CoV-2 booster vaccination. It is possible that the enhanced immune response after booster vaccination and presence of HLA-DR^+^ monocytes could be responsible for triggering the production of the observed MPO- and PR3-ANCA autoantibodies. Additionally, we conducted a systematic review of de novo AAV after SARS-CoV-2 vaccination describing their clinical manifestations in temporal association with SARS-CoV-2 vaccination, ANCA subtype, and treatment regimens. In light of a hundred million individuals being booster vaccinated for SARS-CoV-2 worldwide, a potential causal association with AAV may result in a considerable subset of cases with potential severe complications.

## 1. Introduction

As the coronavirus disease 2019 (COVID-19) pandemic is ongoing, and new variants of severe acute respiratory syndrome coronavirus type 2 (SARS-CoV-2) are emerging, vaccines are needed to protect individuals at high risk of complications and to potentially control disease outbreaks by herd immunity [1]. SARS-CoV-2 has a relatively large genome in comparison with other RNA viruses such as HIV-1 and influenza virus [2,3]. Since the initial SARS-CoV-2 outbreak in Wuhan, the virus has acquired several mutations that affected its infectivity and immunogenicity [4,5]. SARS-CoV-2 variants have been the focus of extensive research due to their rapid spread and high infectivity [6,7]. These include the Alpha variant (B.1.1.7/501Y.V1), the Beta variant (B.1.351/501Y.V2), the Gamma variant (P.1), and the Delta variant (B.1.617.2) [8]. As SARS-CoV-2 vaccines are deployed globally, large clinical trials showed that the SARS-CoV-2 vaccines are safe and effective [9]. Surveillance of rare safety issues related to these vaccines is progressing, since more granular data emerged regarding adverse events due to SARS-CoV-2 vaccines during post-marketing surveillance [1]. Due to the enhancement of the immune response by SARS-CoV-2 vaccination, rare and serious adverse effects have also been reported. These include vaccine-induced immune thrombocytopenia and thrombosis (VITT) and immune-mediated myocarditis in association with the use of viral vector vaccines and mRNA vaccines [10,11,12]. In addition, the new onset of antineutrophil cytoplasmic antibody (ANCA)-associated vasculitis (AAV) is increasingly recognized in association with SARS-CoV-2 vaccines [13]. However, the molecular mechanisms contributing to AAV onset remain elusive. Previous studies suggested that monocytes upregulate major histocompatibility complex (MHC) II cell surface receptor human leukocyte antigen receptor (HLA-DR) molecules in granulomatosis with polyangiitis (GPA) patients with proteinase 3 (PR3-) and myeloperoxidase (MPO-) ANCA seropositivity [14]. It has also been known for a long time that ANCA autoantibodies can target the PR3 and MPO present in the lysosomes of monocytes [15]. These antigens are expressed on the cell surface of cultured monocytes upon activation and can be recognized by the antigen-binding sites of ANCA [16,17]. While insightful about the specific role of monocytes in the pathophysiology of AAV, monocytes seem crucial in the initiation of vascular inflammation and damage [18]. Peripheral blood monocytes are an important source for local macrophage accumulation in parenchymal organs, as evidenced by their presence in early lesions in ANCA-associated glomerulonephritis (GN) [19,20]. Therefore, peripheral monocytes and local macrophages may have an important contribution in the pathophysiology of AAV by modulating inflammation and organ injury. Here, we present a case of new-onset AAV after booster vaccination with the Pfizer-BioNTech SARS-CoV-2 messenger RNA (mRNA) vaccine. Moreover, we provide evidence that the majority of monocytes express HLA-DR in AAV after SARS-CoV-2 booster vaccination.

## 2. Case Description

**Figure 1:**
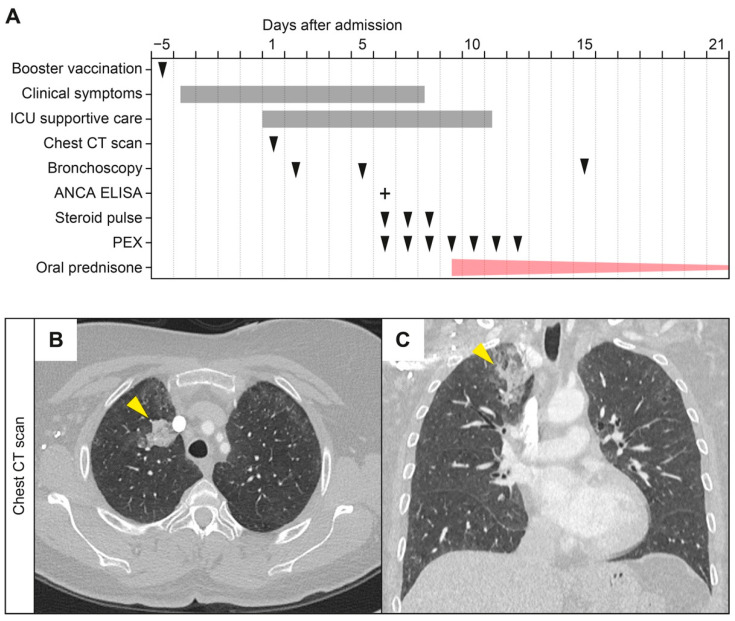
Time course of the case and radiographic findings. (**A**) Time course of booster vaccination, admission, performance of bronchoscopy, chest CT scan, and treatment regimen. (**B**,**C**) Computed tomography of the chest at the time of admission in axial and coronal reformation. At the time of admission, a CT scan confirmed a focal pulmonary hemorrhage in the right upper lobe with focal consolidation (arrowheads) and surrounding ground glass opacities. Furthermore, CT scans revealed subtle ground glass opacities in the anterior upper lobes. Abbreviations: ANCA = antineutrophil cytoplasmic antibody; CT = computed tomography, ELISA = enzyme-linked immunosorbent assay; ICU = intensive care unit; PEX = plasma exchange.

A 57-year-old Caucasian female with a smoking history of 40 pack-years, no medical history of disease, and no documented history of COVID-19 received two doses of Pfizer-BioNTech SARS-CoV-2 vaccines and a recent Pfizer-BioNTech SARS-CoV-2 mRNA booster vaccination. The day thereafter, she developed a pulmonary hemorrhage requiring admission to our emergency department 5 days after booster vaccination (Figure 1A). The vital parameters were stable, and the physical examination was unremarkable. The patient had no allergies and denied illicit drug use. A reverse transcription polymerase chain reaction (RT-PCR) test for SARS-CoV-2 from nasopharyngeal swabs was negative. Laboratory assessments at admission showed only mild leukocytosis of 11,100/µL (reference: 4000–11,000/µL), while the remaining complete blood count, coagulation parameters, C-reactive protein (CRP) serum levels, erythrocyte sedimentation rate (ESR), and urine analysis including microscopy were normal. Due to progressive pulmonary hemorrhage and respiratory failure, the patient was admitted to the intensive care unit (ICU), requiring invasive blood gas monitoring. Chest computed tomography (CT) scans showed ground glass attenuation, consolidation, and thickening of the bronchovascular bundles (Figure 1B,C). A bronchoscopy revealed a hemorrhage localized to the right upper lobe with neutrophilic inflammation in the bronchoalveolar lavage fluid (BALF). Serological testing confirmed the AAV double positive diagnosis for MPO-ANCA (9.9 IU/mL, reference: <3.5 IU/mL) and PR3-ANCA (6.7 IU/mL, reference: <2 IU/mL), while the anti-glomerular basement membrane (anti-GBM) and other ANCA autoantibodies against lactoferrin, elastase, cathepsin G, and bactericidal permeability-increasing protein (BPI) were negative (Table 1). Flow cytometry revealed that the majority of the monocytes expressed HLA-DR on the surface (CD14^+^ HLA-DR^+^: 251 cells/µL, 84.1% of the CD14^+^ population; Table 1). Based on the diagnosis of new-onset AAV presenting with a pulmonary hemorrhage, the patient received a steroid pulse with intravenous methylprednisolone for 3 days (1000 mg per day), oral prednisone daily thereafter (1 mg/kg, 60 mg per day), and a total number of 7 sessions of daily plasma exchange (PEX) with fresh frozen plasma (replacement solution volume: 3000 mL; Figure 1A). Thereafter, the pulmonary hemorrhage improved, and the patient received oral prednisone at 1 mg/kg daily on a tapering regimen until being discharged.

## 3. Systematic Review of the Literature

**Figure 2:**
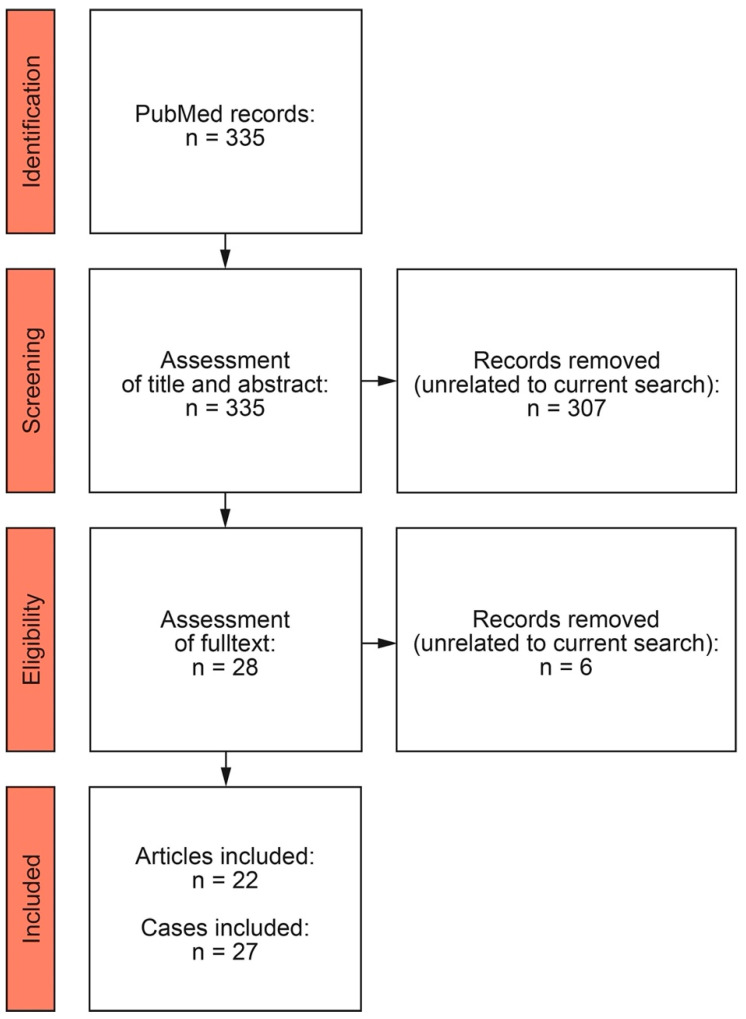
PRISMA flow diagram of the systematic review of the literature.

We conducted a case-based search in PubMed, with the following search (COVID-19 vaccine OR COVID-19 OR COVID-19 vaccination OR SARS-CoV-2 vaccine OR SARS-CoV-2 OR Oxford AstraZeneca OR Moderna OR Pfizer-BioNTech OR Sputnik OR Sinopharm OR BBV152/Covaxin OR Janssen OR CoronaVac OR Novavax) AND (ANCA OR ANCA-associated glomerulonephritis OR ANCA-associated vasculitis OR glomerulonephritis OR MPO-ANCA OR PR3-ANCA OR pauci-immune glomerulonephritis OR de novo vasculitis OR anti-neutrophil cytoplasmic antibody OR antineutrophil cytoplasmic antibody OR myeloperoxidase OR proteinase 3) from 1 January 2020 to 20 April 2022. We included all the case reports published in the English literature of AAV in patients aged ≥ 18 years. Cases were excluded if the AAV developed after SARS-CoV-2 infection, or disease manifestations without ANCA positivity, or if the ANCA status was not reported or untested. The title, abstracts and the full texts of the case reports were individually checked by two authors (E.B. and B.T.) and considered for evaluation if both agreed. Our literature review for articles in English identified 22 articles including 27 cases fulfilling the criteria for de novo AAV reported in temporal association with SARS-CoV-2 vaccination (Figure 2).

Among the cases with de novo AAV, 21 cases received SARS-CoV-2 mRNA vaccination, 3 cases received a viral vector-based vaccine, and 3 cases an inactivated SARS-CoV-2 vaccine (Table 2). AAV was precipitated in 12 patients after the first vaccine dose, and in 15 patients after the second vaccine dose (Table 2). Regarding clinical symptoms, 5 cases presented a hemoptysis, 6 cases presented neurologic symptoms (such as headache, dizziness, blurred vision, or paresthesia), 7 cases presented fever and flu-like symptoms, 6 cases presented fatigue and weakness, 3 presented gastrointestinal symptoms, and 1 case presented cutaneous manifestation (Table 2). Except for 5 patients, all remaining cases presented acute kidney injury (AKI), hematuria, and proteinuria (Table 2). Symptoms developed within one week in 9 cases, and 12 cases developed symptoms within two weeks after SARS-CoV-2 vaccination (Table 2). All AAV cases except of 1 patient were treated with steroid therapy, 5 patients with additional therapeutic plasma exchange (PEX), and 9 patients were further treated with cyclophosphamide, 10 with rituximab, and 2 with both (Table 2). Among the reported cases with de novo AAV after SARS-CoV-2 vaccination, 11 were positive for MPO-ANCA, 7 cases showed PR3-ANCA positivity, 1 case was AAV dual-positive for MPO- and PR3-ANCA, and 2 cases concurrent anti-glomerular basement membrane (anti-GBM) antibodies (Table 2). 

## 4. Discussion

As of yet, our systematic review of the literature revealed 27 published cases of de novo AAV in temporal association with SARS-CoV-2 vaccination. Here, we presented an additional case of a pulmonary hemorrhage due to new-onset AAV that occurred in temporal association with booster vaccination with the Pfizer-BioNTech SARS-CoV-2 mRNA vaccine. Dual-positivity for MPO- and PR3-ANCA has been previously associated with drug-induced AAV, particularly hydralazine, propylthiouracil, and levamisole (typically when in adulterated cocaine) [31,43,44,45]. However, none of these drugs were relevant in the present case. In addition, testing for atypical ANCA autoantigens revealed negative results. In our case, the temporal association between the SARS-CoV-2 booster vaccination and the new onset of dual-positive AAV suggests an immune response to the mRNA vaccine as a potential trigger. Previous studies suggested that monocytes upregulate MHC II cell surface receptor HLA-DR in AAV patients [14]. Moreover, increased HLA-DR^+^ monocytes have already been described in response to influenza vaccination and might be a potential trigger for AAV onset [46]. This is supported by our observation that HLA-DR is present on the surface of most monocytes in this case of AAV in temporal association with SARS-CoV-2 booster vaccination. HLA-DRs are highly efficient molecules which present antigens and initiate immune responses. HLA-DRs are present on B cells, activated T lymphocytes, monocytes or macrophages, dendritic cells, and other non-professional antigen-presenting cells (APCs). In conjunction with the cluster of differentiation 3/T cell receptor (CD3/TCR) complex and CD4 molecules, HLA-DRs are critical for efficient peptide presentation to CD4^+^ T lymphocytes [47]. It is possible that the enhanced immune response after booster vaccination and the presence of HLA-DR^+^ monocytes could be responsible for triggering the production of observed MPO- and PR3-ANCA autoantibodies. Anticipating a hundred million individuals to be booster vaccinated for SARS-CoV-2, a potential causal association with AAV may result in a considerable subset of cases with potential severe complications. Fortunately, treatment of AAV is possible, and insights into the molecular mechanisms underlying the onset of AAV after SARS-CoV-2 (booster) vaccination must be provided by ongoing studies that further enable possible recommendations of early testing if the clinical symptoms are compatible with AAV.

## 5. Conclusions

In summary, we presented here a case of new-onset AAV after booster vaccination with the Pfizer-BioNTech SARS-CoV-2 mRNA vaccine. Moreover, we provided evidence that the majority of monocytes express HLA-DR in AAV after SARS-CoV-2 booster vaccination. It is possible that the enhanced immune response after booster vaccination and the presence of HLA-DR^+^ monocytes could be responsible for triggering the production of the observed MPO- and PR3-ANCA autoantibodies. In light of huge booster vaccination programs for SARS-CoV-2 worldwide, a potential causal association with AAV may result in a considerable subset of cases with potential severe complications. The detection and transparent communication of any adverse events, including rare complications, is important. This is especially relevant since these unusual but severe complications require a specific diagnostic work-up and treatment. Our report aims to sensitize clinicians in the field to this rare but potentially severe complication to encourage the prompt recognition and diagnosis of de novo AAV in timely association with SARS-CoV-2 vaccination, a thorough investigation of possible concurrent triggers, as well as timely treatment once found.

## Figures and Tables

**Table 1:** Key laboratory parameters at admission.

Parameter	Value	Normal Range
Chlamydia pneumoniae IgM (S/CO)	0.02	<0.5
Chlamydia pneumoniae IgA (EIU)	16.01	<8
Chlamydia pneumoniae IgG (EIU)	153.27	<30
Chlamydia psittaci IgM (titer)	<1:12	<1:12
Chlamydia psittaci IgG (titer)	<1:64	<1:64
Chlamydia trachomatis IgA (S/CO)	0.32	<1
Chlamydia trachomatis IgG (S/CO)	0.13	<1
Legionella pneumophila serovar 1–6 IgG (titer)	<1:64	<1:64
Legionella pneumophila serovar 7–14 IgG (titer)	<1:64	<1:64
HIV Ag/Ab (titer)	Neg	Neg
HBs Ag (titer)	Neg	Neg
Anti-HCV (titer)	Neg	Neg
C-reactive protein (mg/L)	3.0	<5.0
Rheumatoid factor (IU/mL)	<10.0	<15.9
Complement C3c (g/L)	1.07	0.82–1.93
Complement C4 (g/L)	0.3	0.15–0.57
ANA IIF (titer)	1:320	<1:100
PR3-ANCA (IU/mL)	6.7	<2.0
MPO-ANCA (IU/mL)	9.9	<3.5
ENA screen (IU/mL)	0.1	<0.7
Anti-GBM (IU/mL)	<0.8	<7.0
LF-lactoferrin (titer)	Neg	Neg
Elastase (titer)	Neg	Neg
Catepsin G (titer)	Neg	Neg
BPI (titer)	Neg	Neg
Anti-ds-DNA (IU/mL)	5.3	<15.0
DSF70 (IU/mL)	<0.6	<7.0
Leukocytes (1000/µL)	11.2	4.0–11.0
Lymphocytes (%)	31.9	20–45
Monocytes (%)	5.1	3–13
Eosinophils (%)	2.5	≤8
Basophils (%)	0.4	≤2
Neutrophils (%)	60.1	40–76
CD14^+^ HLA-DR^+^ (%)	84.1	
CD14^+^ HLA-DR^+^ (cells/µL)	251.0	

Abbreviations: ANA = antinuclear antibody; ANCA = anti-neutrophil cytoplasmic antibody; anti-GBM = anti-glomerular basement membrane; BPI = bactericidal permeability-increasing protein; CD14^+^ = cluster of differentiation 14 positive; ds-DNA = double-stranded-DNA; DSF70 = dense fine-speckled 70; ENA = extractable nuclear antigen; HBsAg = hepatitis B surface antigen; HCV = hepatitis C virus; HIV = human immunodeficiency virus; HLA-DR = human leukocyte antigen DR isotype; IIF = indirect immunofluorescence; MPO = myeloperoxidase; Neg = negative; PR3 = proteinase 3.

**Table 2:** Reported cases of de novo AAV after SARS-CoV-2 vaccination.

Gender	Age	SARS-CoV-2 Vaccine	Onset of Symptoms	Clinical Manifestation	ANCA Positivity	Treatment	Ref.
Female	78	Pfizer-BioNTech (First dose)	16 days	Nausea Vomiting Diarrhoea AKI	MPO-ANCA	Steroids RTX	[21]
Female	79	Pfizer-BioNTech (Second dose)	2 weeks	Weakness Upper thigh pain AKI	MPO-ANCA	Steroids CYC	[22]
Female	29	Pfizer-BioNTech (Second dose)	16 days	AKI	MPO-ANCA	Steroids RTX CYC	[23]
Female	75	Pfizer-BioNTech (First dose)	4 days	Blurred vision	MPO-ANCA	Steroids	[24]
Male	52	Moderna (Second dose)	2 weeks	Headache Weakness AKI	PR3-ANCA	Steroids CYC	[25]
Male	81	Moderna (Second dose)	Not described	Flu-like symptoms AKI	PR3-ANCA	Steroids CYC PEX	[26]
Female	54	Pfizer-BioNTech (Second dose)	2 weeks	Weakness Dizziness Appetite loss AKI	MPO-ANCA	Steroids RTX	[27]
Female	60	Moderna (First dose)	1 day	Fatigue Weight loss Flu-like symptoms	PR3-ANCA	Steroids RTX	[28]
Female	70	Moderna (First dose)	1 week	Dizziness Headache AKI Hemoptysis	MPO-ANCA	Steroids PEX RTX	[29]
Male	58	Moderna (Second dose)	4 days	Nausea Vomiting Weight loss AKI Hemoptysis	PR3-ANCA	Steroids PEX CYC RTX	[30]
Female	37	Pfizer-BioNTech (First dose)	12 days	Erythema Fever	MPO-ANCA PR3-ANCA	Steroids	[31]
Male	63	Oxford AstraZeneca (First dose)	7 days	AKI Hemoptysis	MPO-ANCA	Steroids CYC	[32]
Male	76	Pfizer-BioNTech (Second dose)	11 days	AKI	No subtype	Steroids RTX	[33]
Female	81	Pfizer-BioNTech (Second dose)	2 days	AKI	No subtype	RTX	[33]
Female	76	Moderna (First dose)	5 days	AKI	No subtype	Steroids RTX	[33]
Female	71	Moderna (Second dose)	2 weeks	AKI	No subtype	Steroids RTX	[33]
Female	65	Pfizer-BioNTech (Second dose)	2 weeks	AKI	No subtype	Steroids CYC	[33]
Male	84	Pfizer-BioNTech (Second dose)	1 day	Headache Fever AKI	MPO-ANCA	Steroids	[34]
Male	51	Oxford AstraZeneca (First dose)	15 days	Fever Polyarthritis AKI	PR3-ANCA	Steroids RTX	[35]
Male	23	Moderna (Second dose)	2 weeks	Weakness Fatigue Weight loss AKI	MPO-ANCA Anti-GBM	Not described	[36]
Female	82	Moderna (Second dose)	4 weeks	AKI	MPO-ANCA	Steroids RTX	[37]
Male	58	BBV152/Covaxin (Second dose)	14 days	Hemoptysis Breathlessness AKI	c-ANCA Anti-GBM	Steroids PEX CYC	[38]
Male	45	BBV152/Covaxin (First dose)	12 days	Generalized edema Oliguria Hemoptysis Breathlessness AKI	MPO-ANCA	Steroids PEX CYC	[38]
Female	79	Oxford AstraZeneca (First dose)	35 days	AKI	No subtype	Steroids CYC RTX	[39]
Female	63	Pfizer-BioNTech (First dose)	3 days	Mild fever Right aural fullness Nasal congestion	PR3-ANCA	Steroids CYC	[40]
Female	79	Moderna (Second dose)	<14 days	Back pain Weakness Paresthesia	MPO-ANCA	Steroids	[41]
Female	78	CoronaVac (First dose)	2 weeks	Asthenia Mild fever Mild dry cough AKI	PR3-ANCA	Steroids CYC	[42]

Abbreviations: AKI = acute kidney injury; ANCA = anti-neutrophil cytoplasmic antibody; Anti-GBM = anti-glomerular basement membrane antibody; c-ANCA = cytoplasmic anti-neutrophil cytoplasmic antibody; CYC = cyclophosphamide; MPO = myeloperoxidase; PEX = therapeutic plasma exchange; PR3 = proteinase 3; Ref. = reference; RTX = rituximab.

## Data Availability

Deidentified data are available on reasonable request from the corresponding author.
